# Supplementary material for: Plasma virome and the risk of blood-borne infection in persons with substance use disorder
Source: Nat Commun. 2021 Nov 25;12:6909. doi: 10.1038/s41467-021-26980-8 (PMC8617242; doi:10.1038/s41467-021-26980-8)
Supplement: Supplementary file 1 — Supplementary Information [file 41467_2021_26980_MOESM1_ESM.pdf]

S.Fig.1: Accumulation of non-pathogenic viruses observed among Thai PWID prior to HCV seroconversion

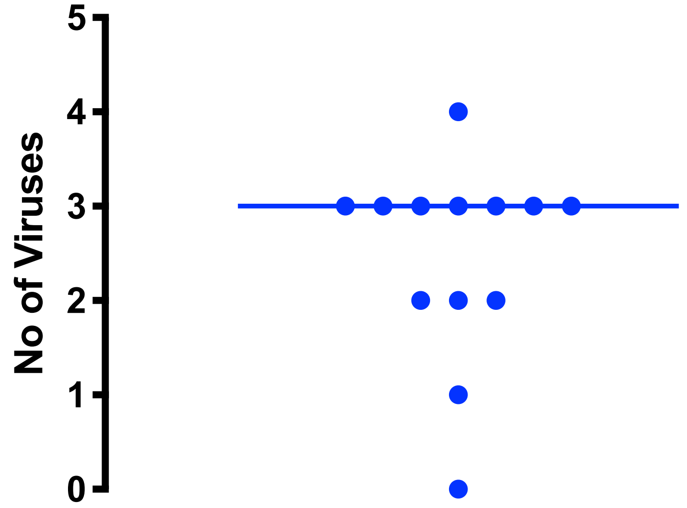

S.Fig.1: Accumulation of non-pathogenic viruses observed among Thai PWID prior to HCV seroconversion: Cross sectional plasma virome assessment of people who inject drugs (PWID) from Chang Mai, Thailand revealed high plasma virome richness among 13 PWID prior to HCV seroconversion. Error bar indicates median.

S. Fig. 2. Phylogenetic analysis of HCV and pegivirus C sequences reveal known and newly recognized drug use pairs

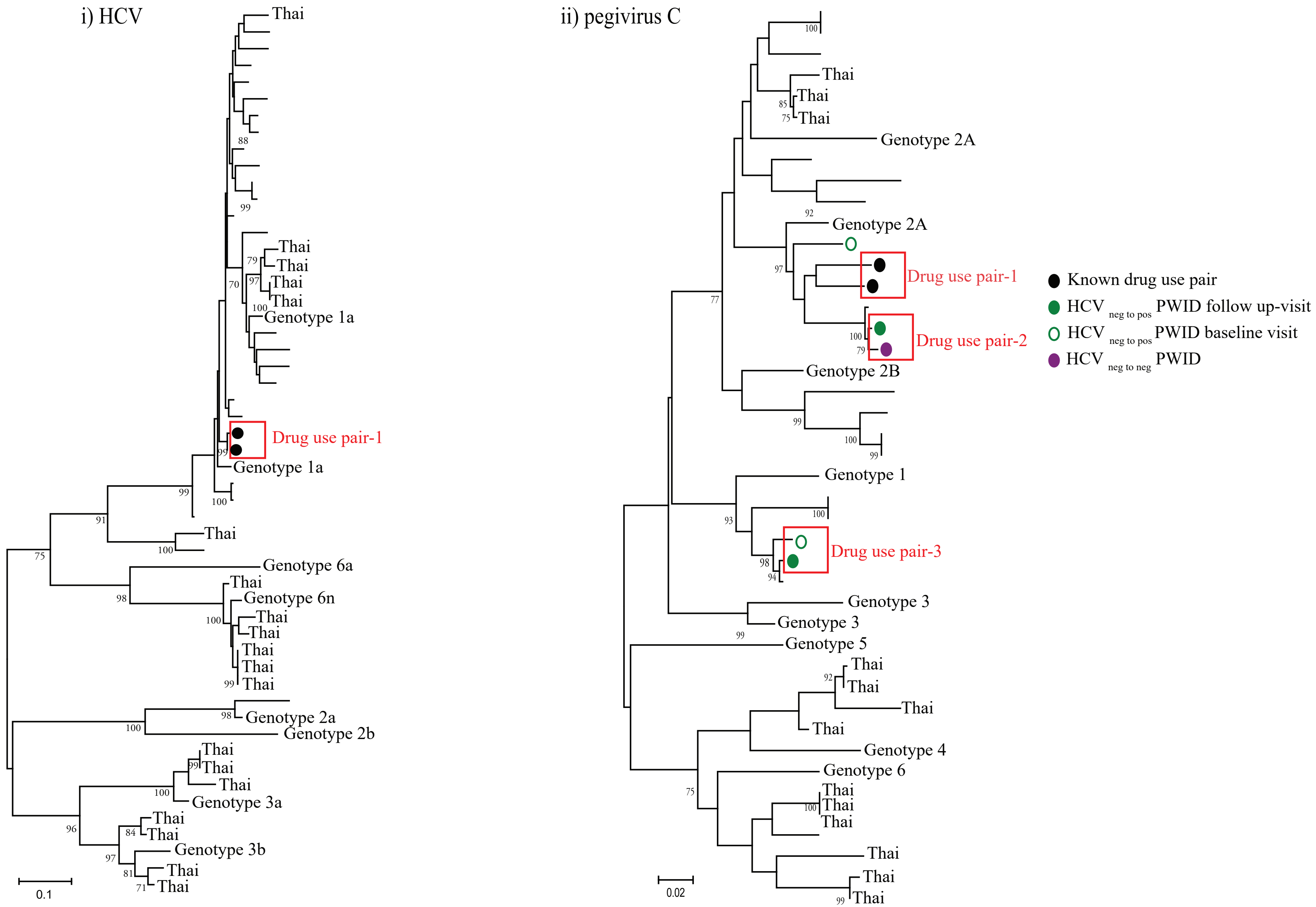

S. Fig. 2. Phylogenetic analysis of HCV and pegivirus C sequences reveal known and newly recognized drug use pairs. Sequences of pegivirus C revealed phylogenetic relationships (Drug use pair 2 and 3) not observed when using only HCV sequences (Drug use pair 1). $HCV_{neg\ to\ pos}$ PWID are HCV seronegative at baseline. The evolutionary history was inferred using the Maximum Likelihood method based on the Tamura-Nei model using MEGA and bootstrap value of 1000. Percentage of trees in which the associated taxa clustered together >75% is shown next to the branches.The tree is drawn to scale, with branch lengths measured in the number of substitutions per site. i) HCV analysis involved 53 nucleotide sequences with 331 positions in the final dataset. ii) Pegivirus C analysis involved 43 nucleotide sequences with 555 positions in the final dataset. All positions containing gaps and missing data were eliminated. Genotype: Genotype reference sequence.

S. Table 1: List of non-pathogenic virome components identified in study participants

i) Discovery panel: $HCV_{neg\ to\ pos}$ PWID (BBAASH 1-20), $HCV_{neg\ to\ neg}$ PWID (BBAASH 21-40).

S.No	Subject ID	Visit -1	Visit-2
1	BBAASH-1	gammatorquevirus	alpha-, gammatorquevirus
2	BBAASH-2	ND	alphatorquevirus
3	BBAASH-3	alphatorquevirus, pegivirus-C	ND
4	BBAASH-4	alphatorquevirus	alpha-,betatorquevirus, pegivirus-C
5	BBAASH-5	ND	alpha-,betatorquevirus,
6	BBAASH-6	alphatorquevirus	alpha-, beta-, gammatorquevirus
7	BBAASH-7	pegivirus-C	ND
8	BBAASH-8	alpha-, beta-, gammatorquevirus	alpha-, betatorquevirus
9	BBAASH-9	alphatorquevirus	ND
10	BBAASH-10	alpha-, betatorquevirus	alpha-, betatorquevirus
11	BBAASH-11	alpha-, beta-, gammatorquevirus, pegivirus-C	alpha-, beta-, gammatorquevirus, pegivirus-C
12	BBAASH-12	ND	pegvirus-C
13	BBAASH-13	ND	alpha-,gammatorquevirus
14	BBAASH-14	alphatorquevirus	alpha-, beta-, gammatorquevirus
15	BBAASH-15	alpha-, beta-, gammatorquevirus,	alpha-, beta-, gammatorquevirus
16	BBAASH-16	alpha-, beta-, gammatorquevirus, pegivirus-C	pegivirus-C
17	BBAASH-17	alpha-, beta-, gammatorquevirus,	alpha-, beta-, gammatorquevirus, pegivirus-C
18	BBAASH-18	alpha-, beta-, gammatorquevirus,	alpha-, beta-, gammatorquevirus, pegivirus-C
19	BBAASH-19	alpha-, beta-, gammatorquevirus, pegivirus-C	ND
20	BBAASH-20	alpha-, beta-, gammatorquevirus,	alpha-, gammatorquevirus
21	BBAASH-21	alpha-, betatorquevirus	alphatorquevirus,
22	BBAASH-22	ND	alpha-, beta-, gammatorquevirus,
23	BBAASH-23	pegivirus-C	alphatorquevirus,
24	BBAASH-24	ND	alphatorquevirus,
25	BBAASH-25	ND	ND
26	BBAASH-26	ND	ND
27	BBAASH-27	ND	ND
28	BBAASH-28	ND	ND
29	BBAASH-29	alphatorquevirus	alphatorquevirus
30	BBAASH-30	alpha-, beta-, gammatorquevirus	alphatorquevirus
31	BBAASH-31	ND	ND
32	BBAASH-32	alphatorquevirus	pegivirus-C
33	BBAASH-33	pegivirus-C	alphatorquevirus,pegivirus-C
34	BBAASH-34	alphatorquevirus,	alphatorquevirus
35	BBAASH-35	ND	pegivirus-C
36	BBAASH-36	alphatorquevirus	ND

37	BBAASH-37	ND	ND
38	BBAASH-38	ND	alphatorquevirus
39	BBAASH-39	ND	alphatorquevirus
40	BBAASH-40	alpha-,gammatorquevirus	ND

ii) Confirmatory panel: $HCV_{neg\ to\ pos}$ PWID (UFO 1-19), $HCV_{neg\ to\ neg}$ PWID (UFO 20-38).

**S.No**	**Subject ID**	**Visit -1**	**Visit-2**
1	UFO-1	alpha-, beta-, gammatorquevirus	alpha-, gammatorquevirus
2	UFO -2	alpha-, beta-, gammatorquevirus	alpha-, gammatorquevirus
3	UFO -3	alpha-, betatorquevirus,pegivirus-C	alpha-, beta-, gammatorquevirus, pegivirus-C
4	UFO -4	alpha-, beta-, gammatorquevirus	alpha-, beta-, gammatorquevirus, pegivirus-C
5	UFO -5	alpha-, beta-, gammatorquevirus	pegivirus-H
6	UFO -6	alpha-, betatorquevirus,pegivirus-C	alpha-, beta-, gammatorquevirus, pegivirus-C
7	UFO -7	alpha-,gammatorquevirus	alpha-, beta-, gammatorquevirus, pegivirus-C
8	UFO -8	alpha-, beta-, gammatorquevirus, pegivirus-C	alpha-, beta-, gammatorquevirus
9	UFO -9	alpha-, betatorquevirus	alphatorquevirus,
10	UFO -10	alpha-, beta-, gammatorquevirus, pegivirus-C	alpha-, beta-, gammatorquevirus, pegivirus-C
11	UFO -11	alpha-, beta-, gammatorquevirus, pegivirus-C	alpha-, beta-, gammatorquevirus,
12	UFO -12	alpha-, gammatorquevirus	alpha-, betatorquevirus,
13	UFO -13	alpha-, betatorquevirus,pegivirus-C	alphatorquevirus, pegivirus-C
14	UFO -14	alpha-, betatorquevirus,pegivirus-C	alphatorquevirus, pegivirus-C
15	UFO -15	alpha-, beta-, gammatorquevirus	alpha-, betatorquevirus
16	UFO -16	alpha-, beta-, gammatorquevirus	alpha-, beta-, gammatorquevirus, pegivirus-C
17	UFO -17	alpha-, betatorquevirus	ND
18	UFO -18	alpha-, betatorquevirus	alphatorquevirus
19	UFO -19	alpha-, beta-, gammatorquevirus	alpha-, gammatorquevirus
20	UFO -20	alpha-, gammatorquevirus, pegivirus-C	betatorquevirus, pegivirus-C
21	UFO -21	alpha-, beta-, gammatorquevirus	alpha-,gammatorquevirus
22	UFO -22	alphatorquevirus, pegivirus-C	alphatorquevirus,
23	UFO -23	ND	alphatorquevirus, pegivirus-C
24	UFO -24	ND	ND
25	UFO -25	ND	ND
26	UFO -26	alpha-, beta-, gammatorquevirus, pegivirus-H	alpha-, betatorquevirus, pegivirus-H
27	UFO -27	alpha-, betatorquevirus, pegivirus-C	Pegivirus-C
28	UFO -28	alpha-, betatorquevirus	ND

29	UFO -29	alpha-, beta-, gammatorquevirus, pegivirus-C	alpha-, gammatorquevirus, pegivirus-C
30	UFO -30	alpha-,gammatorquevirus,	alpha-, beta-, gammatorquevirus
31	UFO -31	alpha-, beta-, gammatorquevirus	alpha-,gammatorquevirus,
32	UFO -32	alpha-, betatorquevirus	alpha-, betatorquevirus
33	UFO -33	alphatorquevirus	ND
34	UFO -34	alphatorquevirus,	alpha-, beta-, gammatorquevirus,
35	UFO -35	alpha-, betatorquevirus, pegivirus-C	alphatorquevirus, pegivirus-C
36	UFO -36	alpha-,gammatorquevirus	alpha-, gammatorquevirus,
37	UFO -37	alpha-, beta-, gammatorquevirus, pegivirus-C	alpha-, gammatorquevirus, pegivirus-C
38	UFO -38	alphatorquevirus, pegivirus-C	pegivirus-C

ii) Control: Non-injection drug users

**S.No**	**Subject ID**	**Visit -1**
1	Control-1	ND
2	Control -2	ND
3	Control -3	ND
4	Control -4	alphatorquevirus
5	Control -5	ND
6	Control -6	alpha-, beta-, gammatorquevirus
7	Control -7	ND
8	Control -8	alphatorquevirus,
9	Control -9	ND
10	Control -10	alpha-, beta-, gammatorquevirus, pegivirus-C
11	Control -11	ND
12	Control -12	ND
13	Control -13	alphatorquevirus
14	Control -14	alpha-, gammatorquevirus
15	Control -15	alpha-,gammatorquevirus
16	Control -16	ND
17	Control -17	ND
18	Control -18	alphatorquevirus
19	Control -19	ND
20	Control -20	ND

ND: Not Detected. $HCV_{neg\ to\ pos}$ PWID: Injection drug users who become HCV seropositive
$HCV_{neg\ to\ pos}$ PWID: Injection drug users who remain HCV seronegative

S. Table 2: PCR primer sequences used for identification of non-pathogenic virome components

a) Anelloviridae

i) Universal

**Primer**	**Nucleotide Sequence (5’ to 3’)**
NG779	ACWKMCGAATGGCTGAGTTT
NG780	RGTGRCGAATGGYWGAGTTT
NG781	CCCKWGCCCGARTTGCCCCT
NG782	AYCTWGCCCGAATTGCCCCT

ii) Alphatorquevirus-specific

NG785	CCCCTTGACTBCGGTGTGTAA

iii) Betatorquevirus -specific

NG791	CTCACCTYSGGCWCCCGCCC
NG792	TTTATGCYGCYAGACGRAGA
NG793	TTTAYCMYGCCAGACGGAGA
NG794	TTTATGCCGCCAGACGRAGG

iv) Gammatorquevirus -specific

NG795	SGABCGAGCGCAGCGAGGAG
NG796	GCCCGARTTGCCCCTAGACC

b) Pegivirus-H

**Primer**	**Nucleotide Sequence (5’ to 3’)**
NS2-3 (FP)	GTGGGACACCTCAACCCTGAAG
NS2-3 (RP)	GGGAAGACAACACCACGATCTGGC

c) Pegivirus-C

**Primer**	**Nucleotide Sequence (5’ to 3’)**
E2_FP	GCCASYTGYACCATAGCYGC
E2_ORP	GCCTCHGCCAGCTTCATCAGRTA
E2_IRP	AAAYACAAARTCCARVAGCARCCA

d) HCV

**Primer**	**Nucleotide Sequence (5’ to 3’)**
CE1_ESP	GCAACAGGGAACCTTCCTGGTTGCTC
CE1_EAP	CGTAGGGGACCAGTTCATCATCAT
CE1_ISP	AACCTTCCTGGTTGCTCTTTCTCTAT
CE1_IAP	GTTCATCATCATATCCCATGC CAT
