# Supplementary material for: Plasma virome and the risk of blood-borne infection in persons with substance use disorder
Source: Nat Commun. 2021 Nov 25;12:6909. doi: 10.1038/s41467-021-26980-8 (PMC8617242; doi:10.1038/s41467-021-26980-8)
Supplement: Supplementary file 3 — Reporting Summary [file 41467_2021_26980_MOESM3_ESM.pdf]

nature portfolio

Corresponding author(s): David L Thomas

Last updated by author(s): Oct 8, 2021

Reporting Summary

Nature Portfolio wishes to improve the reproducibility of the work that we publish. This form provides structure for consistency and transparency in reporting. For further information on Nature Portfolio policies, see our Editorial Policies and the Editorial Policy Checklist.

Statistics

For all statistical analyses, confirm that the following items are present in the figure legend, table legend, main text, or Methods section.

n/a	Confirmed	
☐	☒	The exact sample size (n) for each experimental group/condition, given as a discrete number and unit of measurement
☐	☒	A statement on whether measurements were taken from distinct samples or whether the same sample was measured repeatedly
☐	☒	The statistical test(s) used AND whether they are one- or two-sided<br>*Only common tests should be described solely by name; describe more complex techniques in the Methods section.*
☒	☐	A description of all covariates tested
☒	☐	A description of any assumptions or corrections, such as tests of normality and adjustment for multiple comparisons
☐	☒	A full description of the statistical parameters including central tendency (e.g. means) or other basic estimates (e.g. regression coefficient) AND variation (e.g. standard deviation) or associated estimates of uncertainty (e.g. confidence intervals)
☐	☒	For null hypothesis testing, the test statistic (e.g. F, t, r) with confidence intervals, effect sizes, degrees of freedom and P value noted<br>*Give P values as exact values whenever suitable.*
☒	☐	For Bayesian analysis, information on the choice of priors and Markov chain Monte Carlo settings
☒	☐	For hierarchical and complex designs, identification of the appropriate level for tests and full reporting of outcomes
☒	☐	Estimates of effect sizes (e.g. Cohen's d, Pearson's r), indicating how they were calculated

Our web collection on statistics for biologists contains articles on many of the points above.

Software and code

Policy information about availability of computer code

Data collection	Nanopore sequence data and basecalling was performed on the GridION running 19.12.2 according to protocol.
Data analysis	The software used for data analysis were: i) What's In My Pot (WIMP) v3.2.1, a quantitative analysis tool for nanopore reads, ii) Samtools v1.9 iii) Canu v1.9 to assemble species-specific viral genomes, iv) GraphPad Prism v 9.0.2 for Mac and v) Meg X 10.1.8

For manuscripts utilizing custom algorithms or software that are central to the research but not yet described in published literature, software must be made available to editors and reviewers. We strongly encourage code deposition in a community repository (e.g. GitHub). See the Nature Portfolio guidelines for submitting code & software for further information.

Data

Policy information about availability of data

All manuscripts must include a data availability statement. This statement should provide the following information, where applicable:

- Accession codes, unique identifiers, or web links for publicly available datasets
- A description of any restrictions on data availability
- For clinical datasets or third party data, please ensure that the statement adheres to our policy

TThe sequence data generated in this study have been deposited in the GenBank database under accession code MN938857-MN938890 (pegivirus- C) [https://www.ncbi.nlm.nih.gov/nuccore/
MN938857,MN938858,MN938859,MN938860,MN938861,MN938862,MN938863,MN938864,MN938865,MN938866,MN938867,MN938868,MN938869,MN938870,MN938871,MN938872,MN938873,MN938874,MN938875,MN938876,MN938877,MN938878,MN938879,MN938880,MN938881,MN938882,MN938883,MN938884,MN938885,MN938886,MN938887,MN938888,MN938889,MN938890], MN954475-MN954517 (HCV) [https://www.ncbi.nlm.nih.gov/nuccore/
MN954475,MN954476,MN954477,MN954478,MN954479,MN954480,MN954481,MN954482,MN954483,MN954484,MN954485,MN954486,MN954487,MN95448

8,MN954489,MN954490,MN954491,MN954492,MN954493,MN954494,MN954495,MN954496,MN954497,MN954498,MN954499,MN954500,MN954501,MN954502,MN954503,MN954504,MN954505,MN954506,MN954507,MN954508,MN954509,MN954510,MN954511,MN954512,MN954513,MN954514,MN954515,MN954516,MN954517] , and MZ889122-MZ889127 (alphatorquevirus). The metagenomic dataset generated and analyzed during the current study have been deposited in Sequence Read Archive with the accession code PRJNA764703 [https://www.ncbi.nlm.nih.gov/bioproject/PRJNA764703]. The virome data used in figure 1 are provided in the Supplementary Information/Source Data file.

Field-specific reporting

Please select the one below that is the best fit for your research. If you are not sure, read the appropriate sections before making your selection.

☒ Life sciences ☐ Behavioural & social sciences ☐ Ecological, evolutionary & environmental sciences

For a reference copy of the document with all sections, see nature.com/documents/nr-reporting-summary-flat.pdf

Life sciences study design

All studies must disclose on these points even when the disclosure is negative.

Sample size	For the confirmatory panel a sample size of 20 was determined based on the mean number of circulating viruses observed in plasma,before HCV acquisition, among HCVneg to pos PWID (1.85, 95% CI 1.18-2.52) and HCVneg to neg PWID (0.6, 95% CI 0.21-0.98) and a desired power value of 0.95. Based on sample availability this number was reduced to 19.
Data exclusions	No data was excluded from the study
Replication	The study made use of a confirmatory panel to replicate the observations made in the discovery panel. The discovery panel was drawn from a cohort based in Baltimore,MD,USA while the confirmatory panel had study subjects from San Francisco,CA,USA. A total of 78 biological independent plasma specimens were used - 40 from the discovery and 38 from the confirmatory panel.
Randomization	Randomization was not applicable since the study assessed predefined groups for infection with non-pathogenic virome components.
Blinding	This study used plasma remnants that were collected for other research. Hence, blinding would have made it difficult to identify the appropriate samples for this study.

Reporting for specific materials, systems and methods

We require information from authors about some types of materials, experimental systems and methods used in many studies. Here, indicate whether each material, system or method listed is relevant to your study. If you are not sure if a list item applies to your research, read the appropriate section before selecting a response.

Materials & experimental systems

n/a	Involved in the study	
☒	☐	Antibodies
☒	☐	Eukaryotic cell lines
☒	☐	Palaeontology and archaeology
☒	☐	Animals and other organisms
☐	☒	Human research participants
☒	☐	Clinical data
☒	☐	Dual use research of concern

Methods

n/a	Involved in the study	
☒	☐	ChIP-seq
☒	☐	Flow cytometry
☒	☐	MRI-based neuroimaging

Human research participants

Policy information about studies involving human research participants

Population characteristics	The study samples were drawn from cohorts of injection drug users located in Baltimore (USA), San Francisco (USA), and Chang Mai (Thailand). For the Baltimore participants,the median age in the HCVneg to pos PWID group was 26 years (IQR:6.25) and in the HCVneg to neg PWID group it was 27 years (IQR:4). The gender ratio was 2.33:1 for each group. For the San Francisco participants, the median age in the HCVneg to pos PWID group was 26 years (IQR:4) and in the HCVneg to neg PWID group it was 24 years (IQR:4.5). Each group had a gender ratio of 3.75:1. The Thailand cohort had a gender ratio of 5.50:1 and a median age of 23 years (IQR:17). These cohorts allowed us to study samples before and after HCV acquisition. We also included a control group composed of participants who denied injection drug use. The control group had a gender ratio of 1:1 and median age of 26.5 (IQR:6.77).
Recruitment	The entry criteria for the cohorts are: i) injected drugs in the last 3 months ii) ages 15 to 30 and iii) HCV/HIV negative at the time of enrollment. "PWID were recruited by word of mouth and local advertising. While those who opt to enroll might differ from those who do not, our comparison is among those who enrolled. Therefore, we cannot anticipate any manner in which that bias would affect our primary results. Also, notably, our findings were confirmed in an independent US cohort and were also substantiated by additional controls from Thailand
Ethics oversight	The JHU IRB authorized the research to meet the NIH definition of "not human subjects research" since it used specimens already obtained from person consented for other research and had no patient identifiers linked to the specimens.

Note that full information on the approval of the study protocol must also be provided in the manuscript.
